# Fusobacterium nucleatum and colorectal cancer: From phenomenon to mechanism

**DOI:** 10.3389/fcimb.2022.1020583

**Published:** 2022-11-29

**Authors:** Suwen Ou, Hufei Wang, Yangbao Tao, Kangjia Luo, Jinhua Ye, Songlin Ran, Zilong Guan, Yuliuming Wang, Hanqing Hu, Rui Huang

**Affiliations:** ^1^ Department of Colorectal Surgery, The Second Affiliated Hospital of Harbin Medical University, Harbin, Heilongjiang, China; ^2^ Department of Gastrointestinal Surgery, The Affiliated Hospital of Medical School of Ningbo University, Ningbo, Zhejiang, China; ^3^ Department of General Surgery, The Fourth Affiliated Hospital of Harbin Medical University, Harbin, Heilongjiang, China

**Keywords:** Fusobacterium nucleatum, colorectal cancer, metastasis, metabolic reprogramming, stemness, immune microenvironment, therapy response, prevention

## Abstract

Colorectal cancer(CRC) is the third most frequent malignant tumor. The gut microbiome acts as a vital component of CRC etiology. Fusobacterium nucleatum(Fn) is a key member of colorectal cancer-associated bacteria. But we lack a systematic and in-depth understanding on its role in CRC evolution. In this article, We reviewed the abundance changes and distribution of Fn in CRC occurrence and development, potential effect of Fn in the initiation of CRC, the source of intratumoral Fn and the cause of its tropism to CRC. In addition, We described the mechanism by which Fn promotes the malignant biological behavior of CRC, affects CRC response to therapy, and shapes the tumor immune microenvironment in great detail. Based on the relationship between Fn and CRC, we proposed strategies for CRC prevention and treatment, and discussed the feasibility and limitations of specific cases, to gain insights into further basic and clinical research in the future.

## 1 Introduction

Colorectal cancer (CRC) is a major public health issue. It ranked third in incidence and second in mortality among all cancers in 2020 (Sung et al., 2021). Although both hereditary and environmental risk factors contribute to the development of CRC, genetic variables account for only 12 to 35% of CRC risk factors (Dekker et al., 2019). This indicates that CRC is influenced heavily by environmental risk factors, of which the gut microbiota is a key component (Avril and DePaolo, 2021). Infection with pathogens such as pathogenic *E. coli, Salmonella enterica, toxigenic Bacteroides fragilis, Fusobacterium nucleatum (Fn), Peptostreptococcus anaerobius* and *Helicobacter pylori* has been reported to be associated with the risk of CRC (Tsoi et al., 2017; Dai et al., 2019; Hernández-Luna et al., 2019). Fn is a gram-negative, anaerobic bacillus that doesn’t generate spores (Han, 2006), and classified into four subspecies, including *nucleatum, polymorphum, vincentii(fusiforme)*, and *animalis* (Nie et al., 2015). It exists not only in the human oral cavity but also in the digestive and genitourinary tracts, related with periodontitis (Signat et al., 2011), inflammatory bowel disease (Ohkusa et al., 2003; Minami et al., 2009; Strauss et al., 2011), pancreatic abscess (Brook and Frazier, 1996), hepatic abscess (Athavale et al., 2002; Yoneda et al., 2011), brain abscess (Han et al., 2003; Kai et al., 2008), osteomyelitis (Gregory et al., 2015), pericarditis (Truant et al., 1983), appendicitis (Swidsinski et al., 2011), chorioamnionitis (Altshuler and Hyde, 1988). Although all subspecies of Fn can be detected in CRCs, their intratumoral colonization is heterogeneous. Among them, animalis subspecies is the most prevalent subspecies in CRCs (Ye et al., 2017; Borozan et al., 2022). In addition, in one tumor, colonization by one or two subspecies is commonly observed, whereas colonization by three or four subspecies is detected rarely (Bi et al., 2021). The abundance of Fn is generally elevated in feces, cancer tissues from CRC patients. As a bacterium that colonizes inside CRC, Fn has a great impact on the growth and evolution of CRC. Numerous researches have focused on the role of Fn in CRC in the past decade. Although some breakthroughs have been made in the mechanism of interaction between Fn and CRC, We still lack In-depth and comprehensive understanding of Fn’s role in CRC. In this review, We drew a comprehensive landscape of Fn in the occurrence and development of CRC from the macro and micro perspective, and proposed CRC prevention and treatment strategies based on current understanding of the role of Fn in CRC.

## 2 Temporal and spatial distribution of Fn in CRC

The malignant transformation process of normal intestinal epithelium-precursor lesion-CRC involves two distinct carcinogenesis pathways: the traditional adenoma-carcinoma pathway(70–90%) characterized by Adenomatous polyposis coli (APC) mutation, chromosomal instability, and lack of CpG island hypermethylation; and the alternative/serrated neoplasia pathway(10–20%) characterized by BRAF mutation, chromosomal stability, and high CpG island hypermethylation (Dekker et al., 2019). These pathways reflect a series of genetic and epigenetic events that occur in a logical order. Serrated adenomas/polyps, as precursor lesions of the serrated pathway, are subdivided into sessile serrated adenoma or polyp, traditional serrated adenoma and hyperplastic polyp (JE et al., 2015). Several studies showed that Fn was overabundant in adenomas tissue or stools of adenoma patients compared to healthy people or non-adenoma patients (Kostic et al., 2013; McCoy et al., 2013; Flanagan et al., 2014). Therefore, Fn is already enriched in the lesions (precancerous lesions) before intestinal epithelium cell completing malignant transformation in a subset of patients. Another study showed that hyperplastic polyp and sessile serrated adenomas had a higher prevalence of invasive Fn compared with traditional adenomas(65.7%, 78.8% vs. 28.9% respectively) in proximal colon (Yu et al., 2016). However, the research by Rezasoltani et al. came to a contrary conclusion. In comparison to fecal samples from the normal, hyperplastic polyp, and sessile serrated adenoma groups, traditional adenoma cases, including tubular adenoma and villous/tubuvillous polyp, had a higher amount of Fn. Interestingly, their research showed that there was a positive correlation between the polyp size, dysplasia grade, and the amount of Fn, which were consistent with the finding of Flanagan (Flanagan et al., 2014). In comprehensive consideration, Fn have no preference for carcinogenesis pathways. And the study by Park et al. supports this view because the relative abundance of Fn doesn’t differ between tubular adenoma and sessile serrated adenoma/polyp groups (Park et al., 2016).

Increasing evidence has shown that Fn enrichment is prevalent in CRC. When compared to healthy controls, Fn has a greater overall abundance or presence rate in the fecal microbiota of CRC patients (Kostic et al., 2013; Mira-Pascual et al., 2015; Amitay et al., 2017). Gut microbiota can be classified into two groups: fecal-luminal microbiota and mucosa-associated microbiota (Fung et al., 2014). Mucosa-associated microbiota interacting with intestinal epithelium directly and intimately, is considered more closely related to CRC (Yoon et al., 2017). A study has reported that the amount of Fn in stool is not proportional to the Fn of tissue from the same individual (Flanagan et al., 2014). Therefore, the evidence from the mucosa or tissues is more convincing than stool. The Fn abundance in the tumor tissues of CRC patients is relatively higher than that in mucosal tissues of non-tumor subjects (Xu and Jiang, 2017). Likewise, Fn abundance is substantially higher in the tumor than in neighboring normal mucosal tissue from the same individual (Castellarin et al., 2012; Kostic et al., 2012; Flanagan et al., 2014; Li et al., 2016; Gao et al., 2017; Yamaoka et al., 2018). Interestingly, in the surrounding tissue of CRC, the abundance of Fn is gradually decreased with the distance from the tumor (Cuellar-Gómez et al., 2021; Kono et al., 2021). Fn is also positively associated with the progress of CRC. The study demonstrated that tissues and fecal samples from the patients with invasive CRC(T1b-T3) had a higher Fn abundance than the patients with early CRC(Tis and T1a)(9.65% vs. 0.95%) (Zorron Cheng Tao Pu et al., 2020). Furthermore, another study found that Fn abundance in fecal samples from patients with stage II or III CRC was higher than stage I (Amitay et al., 2017). There is also a positive relationship between Fn and lymph node metastases. The research from Japan showed that the frequency of lymph node metastases in CRC patients with high Fn abundance was higher than low Fn abundance in cancer tissue (59.1% vs. 0%) (Li et al., 2016). These researches above indicates that there is a intense association between Fn and CRC, particularly in the development after initiation.

Overall, CRC in right side has a higher detection rate or relative abundance of Fn than left side (proximal colon> distal colon> rectum) (Salvucci et al., 2021; Borozan et al., 2022). More specifically, the proportion of Fn-high CRC gradually increases from rectum to cecum (Mima et al., 2016a). This may due to the nutritional and environmental preferences of Fn in the gut. According to the data from numerous nations, the presence of Fn in CRC is significantly connected with the molecular characteristics of high microsatellite instability (MSI-H), high CpG island methylator phenotype (CIMP-H), BRAF mutation, TP53 wild type, consensus molecular subtypes 1 (CMS1) (**Table 1**). Among them, the association between Fn and MSI-H is independent of CIMP and BRAF mutation status (Mima et al., 2016b). Interestingly, these are also the molecular characteristics of right-sided CRC (Dekker et al., 2019), and the proportions of CRC with specific molecular features such as MSI-H, CIMP-H, and BRAF and PIK3CA mutations gradually increase along the bowel subsites from rectum to ascending colon (Yamauchi et al., 2012). Whether the presence of Fn leads to these molecular changes, or these molecular features cause the enrichment of Fn in CRC is unknown. Currently, there is no sufficient evidence to support that Fn induces oncogenic mutations in intestinal epithelial cells. Fn gavage, even if the frequency is as high as once a day, was not sufficient to induce colon tumorigenesis in either wild-type mouse model or chronic intestinal inflammation mouse model with IL10 deficiency or T-bet and Rag2 double deficiencies (Kostic et al., 2013; Yu et al., 2015). Fn promotes tumorigenesis only in the presence of oncogenic gene defects, or mutagenic chemical agents AOM or DMH (**Table 2**). Therefore, we tend to hold the thinking that Fn plays a promoting role after the occurrence of key oncogenic mutations in intestinal epithelial cells, rather than directly inducing oncogenic mutations in cells. But research by Lesiów et al. demonstrated that complexes formed by fragments of Fn FomA adhesin with copper significantly stimulated reactive oxygen species production and strong oxidative stress response in CT26 cells (Lesiów et al., 2019). This may trigger DNA damage and further canceration in colon cells. The study by Stokowa-Sołtys et al. provided further direct evidence that copper(II) complexes with fragments of adhesin YadA from Fn possessed DNA-cleaving activity (Stokowa-Sołtys et al., 2022). But these studies lack comparisons with other bacteria. We need to further evaluate the effects of Fn on inducing DNA damage or mutation *in vitro* and *vivo*.

**Table 1:** Molecular characteristics of CRC related to Fn.

Country	Sample size(patients)	Positively correlated molecular features	Negatively correlated molecular features	References
South Korea	246	MSI-H; CIMP-H	/	(Lee et al., 2018)
Japan	511	MLH1 methylation; CIMP-H; MSI-high	/	(Ito et al., 2015)
New Zealand	34	CMS1	/	(Purcell et al., 2017)
Japan and USA	149	CIMP; hMLH1 methylation; CHD7/8 mutation;	TP53 mutation	(Tahara et al., 2014)
USA	1069	MSI-H; MLH1 hypermethylation; CIMP; BRAF mutation	/	(Mima et al., 2016b)
USA	1994	Hypermutated status; MSI; CIMP; ERBB3; POLE	TP53 mutation	(Borozan et al., 2022)
South Africa	55	MSI-H	/	(Viljoen et al., 2015)
Ireland, UK, and USA	1430	CMS1; BRAF mutation;	/	(Salvucci et al., 2021)

MSI-H High microsatellite instability, CIMP CpG island methylator phenotype, CMS1 Consensus molecular subtypes 1, MLH1 mutL homolog 1, ERBB3 erb-b2 receptor tyrosine kinase 3, POLE DNA polymerase epsilon, catalytic subunit, BRAF B-Raf proto-oncogene, serine/threonine kinase.

**Table 2:** Effects of Fn gavage on inflammatory factor and tumorigenesis of colon in various mouse models.

Murine model	Gut microbiota status before Fn inoculation	Breeding environment	Frequency of Fn inoculation	Sample source	Upregulated factors	Non-upregulated factors	Tumorigenesis promotion	References
WT	GF	GF	Once a week	Distal colon	IL17	TNF-a; IL-1β; CXCL1; CXCL2; IFN-γ; IL6; IL10; IL17	/	(Queen et al., 2022)
IL10^-/-^	/	/	Once a day	/	/	/	NO	(Kostic et al., 2013)
T-bet^-/-^ Rag2^-/-^	/	/	Once a day	/	/	/	NO	(Kostic et al., 2013)
Apc^Min/+^	GF	GF	Once a week	Distal colon	/	TNF-a;IL-1β; CXCL1; CXCL2; IFN-γ; IL6; IL10; IL17	NO	(Queen et al., 2022)
Apc^Min/+^	GF	GF	Once a week	Distal colon	/	/	NO	(Tomkovich et al., 2017)
Apc^Min/+^	GF	SPF	Once a week	Distal colon	/	/	NO	(Tomkovich et al., 2017)
Apc^Min/+^IL10^-/-^	GF	SPF	Once a week	Distal colon	/	TNF-a;IL-1β; IFN-γ; IL6; IL22; IL17a	NO	(Tomkovich et al., 2017)
Apc^Min/+^	/	/	Once a day	Colon tumor	IL6; IL8; COX2; TNF-a	IL1b; IL24	YES	(Kostic et al., 2013)
Apc^Min/+^	SPF	SPF	Once a day	/	/	/	YES	(Chen et al., 2020)
Apc^Min/+^	SPF	SPF	Once a day	Serum	MIP3α; IL22; IL21; IL17F	TNF-a; TGFβ1; IFN-γ; IL28; IL23; IL17; IL13; IL12p70; IL6; IL4; IL2; IL1β	YES	(Yang et al., 2017)
Apc^Min/+^	SPF	SPF	Once a day	Colon	IL17F; IL21; IL22; IL23p19; IL31		YES	(Yu et al., 2015)
Apc^Min/+^	SPF	SPF	D0, D14, D18-28*	Colonic epithelium and lamina propria	IL17a	IL6; IL8; TNF; CCL2; COX-2; IL23p19	YES	(Brennan et al., 2021)
ASF WT	a community of eight murine-isolated bacterial strains	GF	Once at the 8th week and an additional two weeks before sacrifice	Colonic lamina propria	IL17a; IL23p19	/	/	(Brennan et al., 2021)
AOM/DSS	SPF	SPF	Twice per DSS-water cycle	Serum	IL1β; IL6	/	YES	(Yu et al., 2020)
AOM	GF	GF	Once	/	/	/	YES	(Ternes et al., 2022)
AOM	SPF	SPF	Twice per DSS-water cycle	Serum	/	IL1β; IL6	NO	(Yu et al., 2020)
AOM/DSS	SPF	SPF	Once every 2 days	Colon tumor	CCL20	/	YES	(Xu et al., 2021)
AOM/DSS	GF	SPF	Once a day	/	/	/	YES	(Kong et al., 2021)
DMH	SPF	SPF	Once a day	Colon	IL17F; IL21; IL22; IL23p19; IL31	MIP3α; IL33	YES	(Yu et al., 2015)

DMH, 1,2-dimethylhydrazine; AOM, azoxymethane; DSS, dextran sodium sulfate; GF, germ-free; SPF, specific-pathogen-free; WT, wild type; IL10^-/-^, IL10 deficiency; T-bet^-/-^Rag2^-/-^, T-bet and Rag2 double deficiencies; Apc^Min/+^, Apc deficiency; Apc^Min/+^IL10^-/-^, Apc^Min/+^ and IL10^-/-^ double deficiencies.

## 3 Ability of Fn to colonize normal gut

To determine whether Fn can induce the transition from normal mucosa to adenoma, we need to assess the ability of Fn to colonize the gut. Fn can’t stably colonize the gut of specific-pathogen-free (SPF) mice raised conventionally, even with repeated inoculations (Queen et al., 2022). However, Fn is able to colonize the gut of germ-free (GF) mice for a long time after oral administration. Furthermore, the study by Brennan et al. showed that the strain Fn7-1 could colonize the gut of neonatal mice and ASF mice(colonized with specific eight murine-isolated bacterial strains). Those suggest that it’s easy for Fn to colonize the gut with simple components of microbiota but difficult to colonize the gut with balanced and complex microbiota. As an alien species, Fn may be repelled by native gut flora. In addition, Fn subspecies differs in the ability of colonizing the gut. This emphasizes that Fn is a heterogeneous species. Under what circumstances can Fn colonize the gut with complex microbiota? The study by Tomkovich et al. demonstrated that Fn could colonize the gut of SPF Apc^Min/+^ mice (Tomkovich et al., 2017). Apc^Min/+^ mice with APC gene deficiency, spontaneously develop intestinal tumors in an aberrant crypt foci-adenoma-adenocarcinoma sequence. Disruption of the mucosal barrier allows Fn easily to invade the mucosa. Moreover, adenoma, as an intermediate of CRC formation progress, highly expresses Gal-GalNAc, which is recognized and bound by Fap2 of Fn (Abed et al., 2016). Consistent with this, Fn is enriched in tumor tissue compared to adjacent normal tissue in Fn-fed Apc^Min/+^ mice (Kostic et al., 2013). In addition, Fn has been reported to be isolated more frequently from mucosal biopsy samples of inflammatory bowel disease patients compared with those from non-inflammatory bowel disease controls (Strauss et al., 2011). Therefore, we infer that Fn can’t stably colonize the normal gut harboring balanced complex microbiota, unless the normal mucosal structure is disrupted, the normal flora is disturbed, or inflammation or dysplasia occurs. These changes may provide Fn with competitor inhibition, more attachment niches, and a more suitable nutrient environment in the gut.

## 4 Oral Fn translocate to CRC *via* two routes

Fn in CRC is thought to originated from the mouth cavity since it is the primary resident member of the human oral microbiota and is rarely seen in the gut (Dewhirst et al., 2010; Faust et al., 2012; Human Microbiome Project Consortium, 2012). This view is further confirmed by Komiya and Abed. The study by Komiya et al. showed that identical strains were detected in more than 40% of CRC patients’ tumors and saliva specimens (Komiya et al., 2019). The study by Abed et al. confirmed the fairly close evolutionary relationship between oral and matched tumor isolates by genome sequence analysis (Abed et al., 2020). Oral Fn can translocate to CRC through two routes. One is the oral cavity-circulatory system-CRC, and the other is the oral cavity-alimentary tract-CRC. In humans, transient bacteremia is frequent during periodontal disease, with bacterial burdens exceeding 104 bacteria/ml blood 15 minutes after tooth brushing (Ashare et al., 2009). The studies by Abed et al. demonstrated that blood-borne Fn can colonize the tumor of the CT26 and MC38 mouse orthotopic CRC models (Abed et al., 2016; Abed et al., 2020). Therefore, oral Fn can enter the circulatory system through broken gums and eventually reach the tumor. In addition, Fn can also translocate to the gut where the tumor is located *via* the digestive tract after swallowing, and then invade the tumor through the damaged mucosa. This route has been confirmed by multiple preclinical studies (Kostic et al., 2013; Wu et al., 2018).

## 5 Affinity molecule mediate Fn’s tropism to CRC

Fn prefers localizing colorectal adenocarcinoma. This phenomenon is partly attributed to the high expression of polysaccharide D-galactose-β(1-3)-N-acetyl-D-galactosamine (Gal-GalNAc) in CRC cells (Abed et al., 2016). The level of Gal-GalNAc is higher in colorectal adenocarcinomas cells compared with normal cells in adjacent tissue. The surface protein Fap2 of Fn is a galactose-binding lectin and has a high affinity to Gal-GalNAc. The attachment of Fn to CRC cell was mediated by the recognization-combination of Gal-GalNAc and Fap2. After O-glycanase treatment or using Fn strains with inactivated Fap2 mutants, the attachment is reduced. The adhesion of Fn to CRC cell is validated not only *in vitro* but also *in vivo* (orthotopic rectal CT26 adenocarcinoma mouse model and Apc^Min/+^ mice model) (Abed et al., 2016). Except for adhesion, Fap2 also mediates intracellular invasion of Fn to CRC cells (Casasanta et al., 2020). Another factor that promotes the enrichment of Fn in CRC is the adhesin FadAc. FadA is a virulence factor of Fn. It appears in two types: secreted mature FadA (mFadA) and non-secreted intact pre-FadA. Neither mFadA nor pre-FadA can bind to E-cadherin alone. Only the complex FadAc formed by mFadA and pre-FadA has binding activity. The surface FadAc presented by Fn has a high affinity to E-cadherin on the surface of CRC cells. The interaction of FadAc and E-cadherin is critical for Fn’s attachment and invasion (Rubinstein et al., 2013). The ability of Fn to bind and invade cells is severely impaired by either deletion of FadA of Fn or downregulation of E-cadherin on CRC cells. However, E-cadherin is expressed in various types of cells. The affinity of FadAc for E-cadherin can’t fully explain Fn’s tropism to CRC. Fap2 and FadA are likely to play a synergistic role in the CRC enrichment of Fn. The binding of Fap2 and Gal-GalNAc allows Fn to selectively localize to CRC. Then, the combination of FadA and E-cadherin strengthens the attachment of Fn to CRC cells. Invasion to CRC cells mediated by FadAc allows Fn to hide within CRC cells, thereby avoiding clearance by the immune system.

## 6 Inflammation associated tumorigenicity of Fn in mouse model

Inflammation-induced genetic and epigenetic alterations are important aspects of CRC initiation, particularly in colitis-associated CRC. Studies on whether Fn promotes tumorigenesis by inducing inflammation are inconsistent (**Table 2**). Multiple studies have shown that in wild-type murine model, Fn gavage failed to trigger colitis characterized by increased histological colitis score, colon shortening and upregulation of inflammatory factors (Brennan et al., 2021; Queen et al., 2022). Besides, Fn was inferior to pks+ Escherichia coli and failed to promote colon inflammatory factors expression and colon tumorigenesis in GF/SPF Apc^Min/+^mice and SPF Apc^Min/+^IL10^-/-^ mice (Tomkovich et al., 2017; Queen et al., 2022). Differently, other two studies showed that Fn successfully promoted colon tumorigenesis and the expression of a cluster of the inflammatory gene, such as IL6, IL8, COX2, TNF-a, MIP3a(also named CCL20), IL22, IL21, IL17F in SPF Apc^Min/+^ mice (Kostic et al., 2013; Yang et al., 2017). It should be noted that the gavage frequency in the latter two studies was higher than the former two studies(once a day vs. twice a week). Since Fn is difficult to stably colonize the normal gut of mice, high-frequency oral administration may increase the colonization and abundance of Fn in the mucosa. Therefore, we infer that Fn-induced colitis and tumorigenesis may be dose-dependent. Furthermore, these studies were conducted in different places, and the mice lived in different environments. The differences of gut microbiota may cause inconsistence of the research results. Fn-induced inflammation may also require a specific consortium of cross-communicating or synergistic bacteria. Oral feeding of Fn was shown to be tumorigenic in the Azoxymethane(AOM)-Dextran sodium sulfate(DSS) murine model(a colitis-associated CRC model). However, the tumorigenic effect of Fn was abolished without pro-colitis agent DSS, indicating that Fn and inflammation induced by other factors exert a synergistic effect in colon tumorigenesis (Yu et al., 2020).

## 7 Fn accelerates CRC proliferation and metastasis

**Figure 1:**
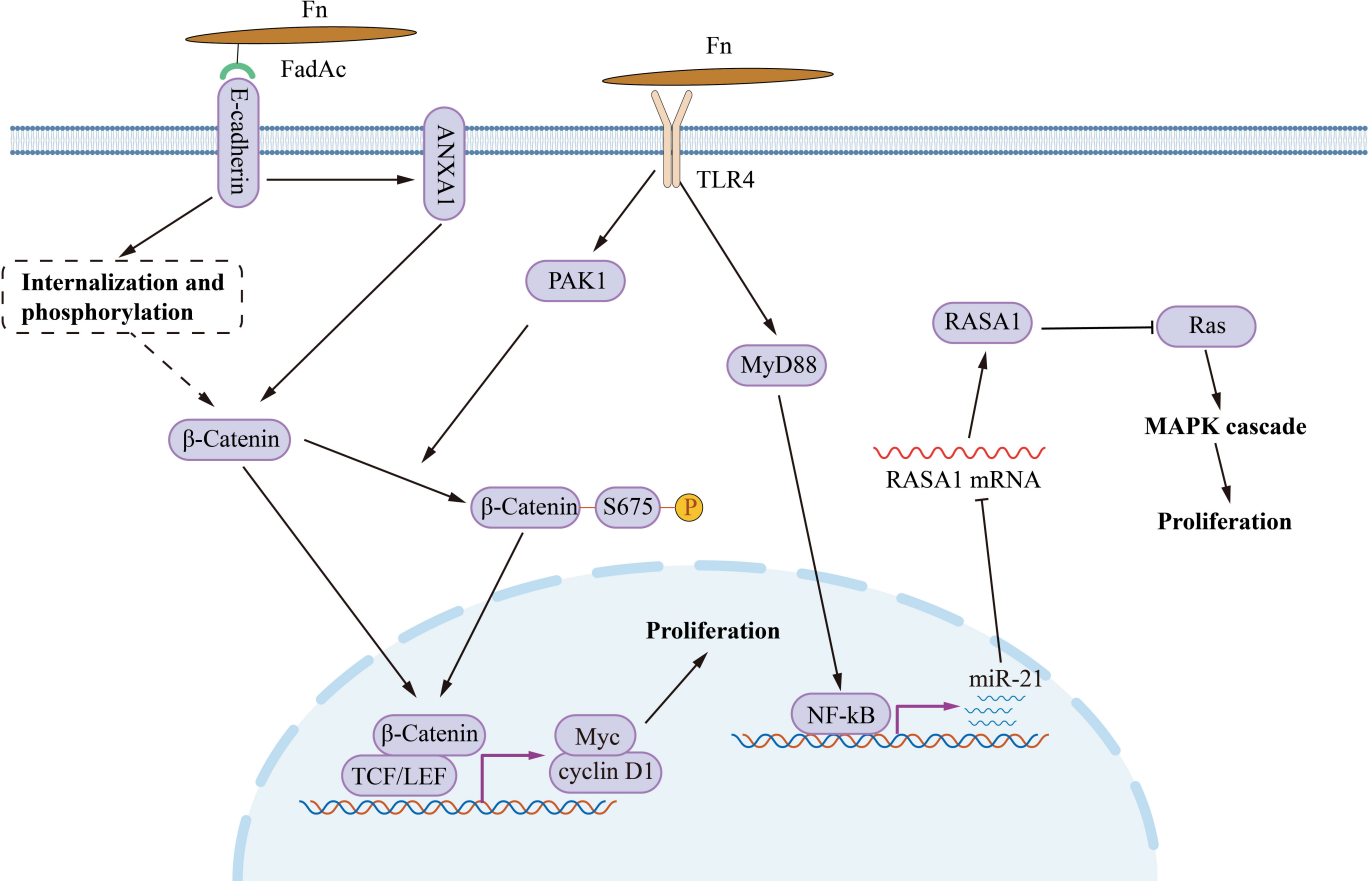
The mechanism of Fn promotes CRC cell proliferation. Fn adhesin FadAc binding to E-cadherin on CRC cell membrane leads to E-cadherin phosphorylation and internalization, enhancing the cytoplasmic accumulation and nuclear translocation of β-catenin. FadAc also up-regulates Annexin A1 *via* E-cadherin, activating β-catenin signaling. Fn lipopolysaccharides activate PAK1 through TLR4, and phosphorylates β-catenin at Ser675 by activating PAK1 (p-PAK1). Phosphorylation increases the nuclear accumulate of β-catenin. Nuclear β-catenin activates the transcription of downstream genes, such as CCND1, and MYC, and then promotes CRC cell proliferation. Fn activates TLR4/MYD88/NF-κB signaling to promotes miR-21 transcription. miR-21 activates the MAPK cascade by inhibiting RASA1, subsequently accelerates cancer cell proliferation.

Fn infection aggravates the malignancy proliferation of CRC (**Figure 1**). In CRC cells HCT116, LoVo, HT29 and SW480, incubating with Fn activates TLR4/MYD88/NF-κB signaling to promotes miR-21 transcription (Yang et al., 2017). Upregulated miR-21 inhibits the expression of RASA1, subsequently accelerates cancer cell proliferation *via* activating the MAPK cascade. Except for MAPK, abnormal activation of the Wnt/β-catenin signal is the initiation factor of most CRC (Dekker et al., 2019). Fn adhesin FadAc binding to E-cadherin on the CRC cell membrane leads to E-cadherin phosphorylation and internalization, leading to the cytoplasmic accumulation and nuclear translocation of β-catenin (Rubinstein et al., 2013). Increased nuclear translocation of β-catenin activates the transcription of downstream genes, such as CCND1 and MYC, and promots CRC cell proliferation. Besides, Annexin A1 is an important CRC growth stimulator, with increased expression in CRC cells (Rubinstein et al., 2019). The Fn FadAc up-regulates Annexin A1 through E-cadherin, activating β-catenin signaling, finally accelerating the proliferation of CRC cells (Rubinstein et al., 2019). P21-activated kinase 1 (PAK1) is a member of the PAK family of serine/threonine kinases. Fn and its lipopolysaccharides activate PAK1 through TLR4, and activated PAK1 (p-PAK1) phosphorylates β-catenin at Ser675. β-catenin phosphorylation enhances its transcriptional activity, and activates the expression of CCND1 and MYC (Chen et al., 2017).The activation of TLR4/PAK1/β-catenin cascade was also observed in Fn-gavaged Apc^Min/+^ mice (Wu et al., 2018).

**Figure 2:**
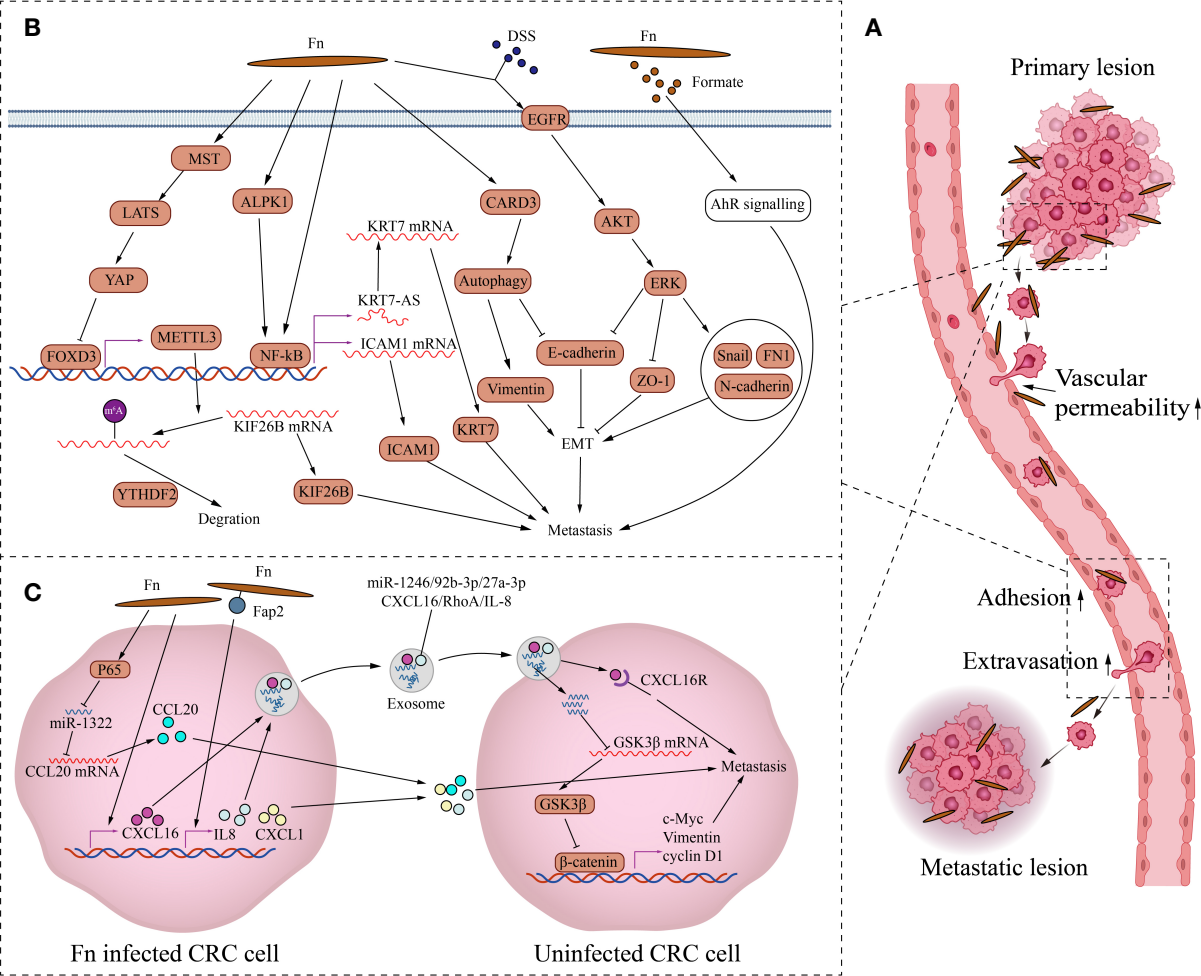
The mechanism of Fn promoting CRC metastasis. **(A)** This is the schematic diagram of CRC distant metastasis. Fn exists in primary and metastatic lesions. Fn infection can increase vascular permeability, CRC cells attachment to endothelial cells and extravasation to promote metastasis. **(B)** Fn promotes metastasis by regulating intercellular signals. Fn inhibits transcription factor FOXD3 through activating MST/LATS/YAP axis axis. The downregulation of FOXD3 inhibits the expression of its target gene METTL3, finally increases the level of KIF26B by reducing the YTHDF2-dependent mRNA degradation, to promote metastasis. Fn activates NFKB through ALPK1 or other paths, Activated NFKB promotes CRC metastasis by KRT7-AS/KRT7, and also by increasing ICAM1. Fn induces epithelial mesenchymal transformation of CRC cells to promote metastasis by activating CARD3 mediated autophagy, and also by activating EGFR/AKT/ERK siganaling. Formate, metabolite of Fn promotes the metastatic dissemination by activating the AhR signal. **(C)** Fn promotes metastasis by activating intracellular signals. Fn infection induces the expression and secretion of cytokines CXCL1 and IL-8, which promote metastasis of non-Fn-exposed CRC cells. Fn upregulates CCL20 by activating NF-κB/miR-1322 signaling, and promotes metastasis of CRC. Fn infection increases CRC cells the release of exosome full of miR-1246/27a-3p/92b-3p and CXCL16/IL-8/RhoA to promotes metastasis of other CRC cells.

Fn promotes metastasis by regulating signaling molecules between CRC cells (**Figure 2**). Fn’s direct adhesion and invasion to CRC cells induces the expression and secretion of cytokines CXCL1 and IL-8, which promote chemotactic migration of non-Fn-exposed CRC cells (Casasanta et al., 2020). Fn outer membrane adhesins Fap2 are critical for the upregulation and secretion of IL-8 and CXCL1. Fn-induced cell migration can be reduced by blocking Fn-host-cell binding and internalization with Fap2 knockout, galactose sugars, l-arginine, or neutralizing antibody. However, exhaustion of the cytokines IL-8 and CXCL1 in the culture medium can only partially reverse CRC migration, indicating that other factors or mechanisms may contribute to CRC cell migration. CCL20 is also involved in Fn-mediated CRC metastasis. Fn upregulates CCL20 by activating NF-κB/miR-1322 signaling, and promotes the migration and metastasis of CRC cells (Xu et al., 2021). Fn infection increases CRC cells exosome release which is rich in miR-1246/27a-3p/92b-3p and CXCL16/IL-8/RhoA (Guo et al., 2020).The exosomes delivers these signal molecules from Fn-infected cells into non-infected cells. Among them, miR-1246/27a-3p/92b-3p suppress GSK3β expression by directly targeting the mRNA 3’-untranslated region, finally activating the Wnt/β-catenin pathway, promoting epithelial-mesenchymal transition and metastasis of CRC cells. CXCL16 in exosomes also plays an important role in promoting the migration of recipient CRC cells through the CXCL16/CXCR6 axis. In summary, Fn-infected CRC cells can transmit information molecules to uninfected CRC cells in two ways, direct secretion and exosomes to promote migration and metastasis.

Fn also promotes CRC metastasis by activating intracellular signaling (**Figure 2**). As a structural fibrous protein, keratin 7(KRT7) plays a role in maintaining cell structural integrity and promoting cell motility (Helfand et al., 2004). LncRNA KRT7-AS is a single antisense RNA transcribed from the KRT7 negative strand. Fn infection increases its transcription level of KRT7-AS by activating the NF-κB pathway in CRC cells (Chen et al., 2020). Increased KRT7-AS transcript promotes CRC metastasis by enhancing KRT7 mRNA stability and translation. Besides, Fn infection promotes CRC metastasis by activating the autophagy pathway *via* upregulating the receptor-interacting protein CARD3 (Chen et al., 2020). The upregulation of CARD3 leads to activation of autophagy, characterized by upregulation of LC3-II, Beclin1, downregulation of P62, and increased formation of autophagosomes. The upregulation of CARD3 also leads to elevated epithelial-mesenchymal transition (EMT) and metastasis ability of CRC cells characterized by upregulation of Vimentin and downregulation of E-cadherin. And these effects are weakened by autophagy inhibitor chloroquine. DSS is a well-known colitis-inducing agent. DSS and Fn co-treatment increased the motility and EMT characteristics of CRC cells compared with Fn infection alone (Yu et al., 2020). The potential mechanism is that Fn and DSS stimulate EMT through epidermal growth factor receptor (EGFR) activation. The co-treatment of CRC cells with Fn and DSS increases the phosphorylated levels of EGFR, inducing the activation of downstream effector kinases, including protein kinase B (AKT) and extracellular signal-regulated kinase (ERK), finally leading to the upregulation of EMT transcription factor Snail and mesenchymal markers fibronectin and N-cadherin, the downregulation of epithelial markers E-cadherin and ZO-1. Fn also promotes tumor formation and the expression of EMT markers in the AOM-DSS murine model. In addition, N6-methyladenosine (m6A), an important epitranscriptome modification, affects various fundamental biological processes by regulating mRNA (Patil et al., 2018). By inhibiting the Hippo pathway, Fn treatment activates YAP signaling in CRC cells (Chen et al., 2022). YAP nuclear translocation suppresses the expression of the downstream target gene METTL3 by inhibiting the transcription of FOXD3,which is the transcription factor of METTL3. Downregulation of METTL3, a major m6A methyltransferase, reduces the YTHDF2-dependent KIF26B mRNA degradation and promotes KIF26B expression by reducing the m6A modification of KIF26B mRNA, ultimately promoting CRC metastasis. To spread to other sites, tumor cells need to undergo a process of infiltration, adhesion, and extravasation (Reymond et al., 2013). The metastatic spread is an inefficient process because only a small fraction of cancer cells in the primary tumor have the potential to form metastases. Even if a patient has cancer cells in their blood, distant metastases may not necessarily occur. Adhesion of cancer cells to endothelial cells and transendothelial invasion of cancer cells are critical steps in the metastatic process. Intercellular adhesion molecule 1 (ICAM1) is a transmembrane protein that participates in direct interactions between cells, including cancer cell and vascular endothelial cell (Wai Wong et al., 2012). Fn upregulates ICAM1 to promote CRC cells attachment to endothelial cells and extravasation by activating the alpha-kinase 1(ALPK1)/NF-κB pathway, ultimately facilitating the distant metastasis of CRC (Zhang et al., 2022). In addition, Fn-derived metabolite formate drives CRC cells’ metastatic dissemination by triggering aryl hydrocarbon receptor (AhR) signaling (Ternes et al., 2022).

Tumor metastasis is also related to the destruction of the vascular endothelial barrier. Fn adhesion to endothelial cells requires the affinity between Vascular endothelial-cadherin (VE-cadherin) and FadA on Fn. Fn adhesion to endothelial cells causes VE-cadherin to be repositioned away from cell-cell junctions, increasing permeability between endothelial cells and allowing bacteria to pass through loosened junctions (Fardini et al., 2011). Fn infection also reduces CD31 expression in endothelial cells, implying decreased cell-cell contact and increased vascular permeability (Mendes et al., 2016). There is no doubt that the destruction of the vascular endothelial barrier not only contributes to the invasion of Fn but also contributes to the hematogenous metastasis of cancer cells. Overall, increased vascular endothelial permeability, enhanced attachment of cancer cell to vascular endothelium, and enhanced epithelial-mesenchymal transition and motility of tumor cells by Fn jointly promote the metastasis of CRC cells.

## 8 Fn enhances stemness of CRC cell

Cancer stem cells (CSCs), a subset of tumor cells, are considered to be responsible for tumor initiation, progression, resistance to chemotherapy, and recurrence (Ishizawa et al., 2010; Nassar and Blanpain, 2016). Compared with other tumor cells, CSCs have the following characteristics: unlimited self-renewal ability, stronger spheroidization ability *in vitro* and tumorigenic ability and reconstruction of tumor heterogeneity *in vivo*, high expression of stem cell marker, epithelial-mesenchymal transition, radiotherapy and chemotherapy resistance (Batlle and Clevers, 2017). Both CSCs and non-CSCs are plastic. CSCs can undergo a phenotypic transition in response to appropriate stimuli. In 2019, we proposed the conjecture that Fn may promote CRC drug resistance by increasing the proportion of CSCs in CRC (Luo et al., 2019). Consistent with our hypothesis, studies suggested that Fn infection increased the expression of stem cell marker CD44 and CD133, and the ability of spheroid formation in CRC cells (Yu et al., 2020; Wang et al., 2020).The research by Liu et al. not only confirmed the Fn-induced transformation of the stem cell-like phenotype of CRC cells but also further elucidated the mechanism (Liu et al., 2022). Numb is a cell fate determinant that promotes CSCs differentiation by inhibiting Notch signaling, thus regarded as a suppressor in various cancers (Colaluca et al., 2008). In colorectal cancer stem cells(CCSCs), Fn reduces lipid accumulation by promoting fatty acid oxidation and provides energy for CCSCs self-renewal and proliferation. On the other hand, Fn increases lipid droplet in non-CCSCs by promoting fatty acid and triglyceride synthesis. Accumulated lipid droplets activate Notch signaling by recruiting Numb and E3 ubiquitin-protein ligase MDM2 to promote the ubiquitinated degradation of Numb. Fn infection inhibits CCSCs differentiation, and allows non-CCSCs to acquire stemness features. In addition to Fn itself, its metabolite formate can also induce stemness in CRC cells. Treatment of mice bearing CRC xenografts with formate increases the CSC markers Aldehyde dehydrogenase(ALDH), CD44, and octamer-binding transcription factor 4(OCT4) expression in tumors (Ternes et al., 2022). The underlying mechanism could be that Fn-derived formate activates the aryl hydrocarbon receptor signaling, because the aryl hydrocarbon receptor is involved in regulating the stemness of cancer cell (Stanford et al., 2016).

## 9 Fn regulates tumor metabolic reprogramming

Metabolic disorder is one of the hallmarks of cancer (Hanahan and Weinberg, 2011). Normal cells use glucose for energy production mainly through the oxidative phosphorylation pathway in the presence of sufficient oxygen. Unlike normal cells, cancer cells process glucose primarily through glycolysis. It is well known that adenosine triphosphate (ATP) production by oxidative phosphorylation is 18 times more than glycolysis. This is not a wise choice from an energy production efficiency standpoint. Cancer cells not only require a lot of energy but also need to synthesize many substances to generate new cells, because cancer cells are constantly proliferating. Increased glycolysis allows more glycolytic intermediates to be diverted into various biosynthetic pathways, including nucleotides, amino acids, and lipids (Vander Heiden et al., 2009). Fn promotes glycolysis in CRC cells by upregulating a key glycolytic enzyme Enolase 1 (ENO1) (Hong et al., 2020). Fn activates transcription of lncRNA ENO1-IT1 by upregulating transcription factor SP1. Elevated ENO1-IT1 directs the histone acetyltransferase KAT7 to its target gene ENO1, increasing the level of acetylation of histone H3 lysine 27(H3K27Ac) in the promoter region. H3K27Ac modification in the promoter region of ENO1 enhanced the transcriptional activity of ENO1 and consequently accelerates the glycolysis and proliferation of CRC cells. In addition, Fn also promotes glucose uptake and glycolytic activity in CRC cells by up-regulating angiopoietin-like 4(ANGPTL4) (Zheng et al., 2021). Fn infection up-regulated the transcription factor HIF1a level, and also enhanced the H3K27Ac level of the HIF1a-binding site in the ANGPTL4 promoter region by down-regulating histone deacetylases. Promoter region H3K27Ac and upregulation of transcription factors synergistically promote ANGPTL4 transcription and expression. The up-regulation of ANGPTL4 not only promotes glycolysis but also promotes glucose uptake in CRC cells by upregulating the glucose transporter GLUT1. Interestingly, the upregulation of ANGPTL4, which is induced by Fn, promotes Fn intracellular colonization in CRC cells.

Fn regulates lipid metabolism in CRC. Cytochrome P450 2J2 (CYP2J2), a member of the cytochrome P450 superfamily of enzymes, can metabolize linoleic acid to 9,10-epoxyoctadecaenoic acids(9,10-EpOME) and 12,13-epoxyoctadecaenoic acids (12,13-EpOME). EpOMEs have been reported to have cancer-promoting activity in a mouse model of CRC (Wang et al., 2019). CYP2J2 has also been found to be upregulated in a variety of cancers and associated with drug resistance (Karkhanis et al., 2017). Fn infection upregulates CYP2J2 expression in CRC cells *via* TLR4/AKT/Keap1/NRF2 signaling (Kong et al., 2021). NRF2 is a transcription factor of CYP2J2 and promotes its expression. Elevated CYP2J2 increases the production of 12,13-EpOME, which finally accelerates the EMT, metastasis, and development of CRC.

## 10 Fn interacts with tumor immune microenvironment

**Figure 3:**
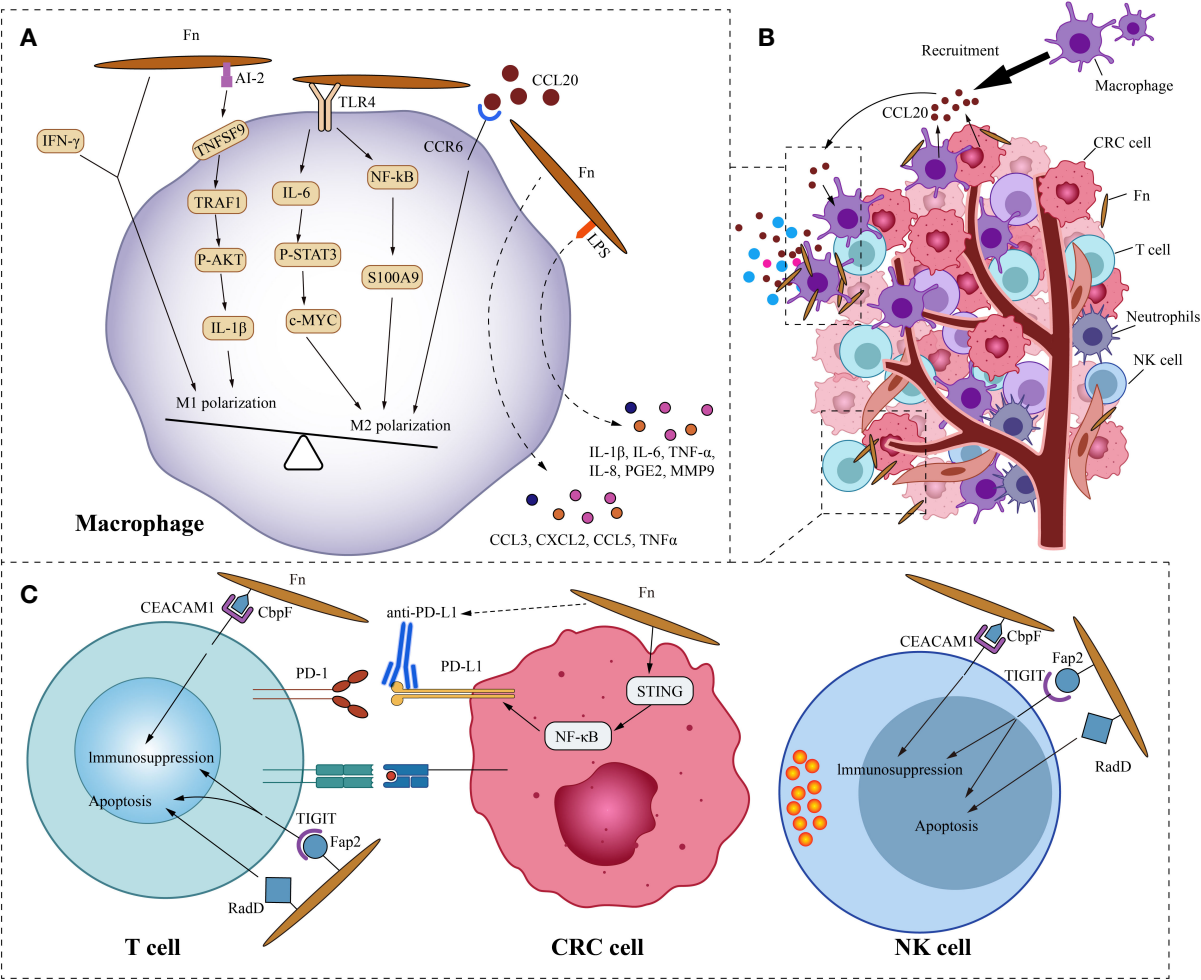
Fn interacts with CRC immune microenvironment. **(A)** This is the schematic diagram of communication of Fn and CRC immune microenvironment. **(B)** In general, Fn induces macrophages to M2 polarization. Fn promotes macrophages M2 polarization by activating TLR4/IL6/p-STAT3/c-Myc and NFKB/S100A9 signals. But some components such as AI-2 of Fn and IFN-γ induced by Fn mediate macrophages MI polarization. CRC cells and macrophages derived CCL20 induced by Fn infection also promotes macrophages M2 polarization. Other molecules derived from macrophages induced by Fn may also have important effects in the immune microenvironment, such as IL-6, IL-1β, TNF-a, IL-8, PGE2 and MMP9. **(C)** Fn binds to the inhibitory receptors TIGIT and CEACAM1 on T cells and NK cells through Fap2 and CbpF, respectively, to mediate their immunosuppression and apoptosis. Fn upregulates the expression of PD-L1 in CRC cells by activating the STING/NFKB axis, and enhances the response of CRC to PD-L1 therapy.

The components of tumor microenvironment (TME), such as immune cells, fibroblasts, signaling molecules, extracellular matrix, and blood vessels, constantly interact with the tumor, affecting its growth and evolution. Fn, as the overabundant intratumoral bacteria in CRC, cross-communicate with the tumor microenvironment. We focus on the crosstalk mechanism of Fn and immune cells (**Figure 3**). In the ApcMin/+ mouse model, Oral feeding of Fn promoted colon tumorigenesis and altered the intratumoral immune microenvironment. Although CD3+CD4+and CD3+CD8+T lymphocytes were not significantly affected, CD11b+ myeloid immune cells in the colon tumor were expanded by Fn. Tumor-associated macrophages(TAMs,especially M2), myeloid-derived suppressor cells(MDSCs), tumor-associated neutrophils, and dendritic cells were enriched in colon tumors of Fn-fed mice (Kostic et al., 2013). Consistently, the presence of Fn was also associated with a higher density of MDSCs in liver metastases of human CRC (Sakamoto et al., 2021). As we know, these components of the tumor immune microenvironment are closely related to tumor progression.

### 10.1 Fn and monocyte/macrophage polarization

Macrophages, as one of the most abundant infiltrating leukocytes in TME, play an important role in cancer progression by stimulating proliferation, metastasis, and angiogenesis while creating an immunosuppressive environment (Cendrowicz et al., 2021). Two retrospective studies from Korea showed that intratumoral Fn was positively correlated with macrophage infiltration and M2 polarization in tumors (Park et al., 2017; Lee et al., 2021). This is consistent with the study by Kostic et al (Kostic et al., 2013). The study by Ye et al. found that Fn and the cytokine C-C motif chemokine ligand 20(CCL20) were significantly increased in CRC compared with normal mucosa (Ye et al., 2017). This implies a potential link between Fn and CCL20. Their further study found that co-culture with Fn induces CCL20 expression in CRC cells and monocytes. They also observed that Fn-induced monocyte migration was partially inhibited by CCL20 neutralizing antibody, suggesting that CCL20 signal is involved in the regulation of monocyte/macrophage motility. Consistent with the findings of Ye, the research by Xu et al. showed that Fn infection stimulated the expression of CCL20 in CRC cells. Moreover, CRC cell-derived CCL20 promoted macrophage migration and M2 polarization *in vitro*, and also promoted intratumoral infiltration of macrophages *in vivo* (Xu et al., 2021). Macrophages are extremely plastic cells that may modify their function dramatically based on the microenvironmental signals in the TME. Based on their diverse functions, macrophages are divided into two opposite subtypes: classically activated macrophages (M1, a pro-inflammation subtype) and alternatively activated macrophages (M2, an anti-inflammation and tumor-promotion subtype) (Wu et al., 2019). Fn not only induces the recruitment of macrophages into the tumor but also regulates the polarization of macrophages. The ability of Fn to induce M2 polarization of macrophages is stronger than *E. coli*. Fn stimulates S100A9 expression *via* TLR4/NF-kB signaling in CRC cells and macrophages (Hu et al., 2021). S100A9 may activate macrophages and M2 polarization in an autocrine or paracrine manner, ultimately promoting the progress of CRC. Similarly, the study by Chen et al. showed that Fn induced macrophage M2 polarization *via* a TLR4/IL-6/p-STAT3/c-MYC cascade (Chen et al., 2018).

However, Fn doesn’t always induce macrophage M2 polarization. Liu and colleagues found that in a mouse model of DSS-induced enteritis, Fn increased the infiltration of M1 macrophages in colon tissue (Liu et al., 2019). Further study of the mechanism found that under the co-stimulation of Fn and IFN-γ, Fn can induce the M1 polarization of mouse bone marrow-derived macrophages, but Fn alone can’t stimulate it. Sakamoto and colleagues found that in the liver metastases of human CRC, the presence of Fn was not significantly correlated with M2 macrophages (Sakamoto et al., 2021). Fn induces robust CCL3, CXCL2, CCL5, and TNFα secretion in mouse macrophages *in vitro (* Casasanta et al., 2020). Unlike Fn-induced CRC cell inflammatory factor secretion, the cytokine secretion of these macrophages does not depend on Fap2 of Fn. CCL3 stimulates lymphocyte recruitment, while CXCL2 promotes angiogenesis in CRC. Grenier et al. found that the lipopolysaccharide of Fn significantly increased the secretion of pro-inflammatory cytokines IL-6, IL-1β, TNF-a, IL-8, and PGE2 in macrophages (Grenier and Grignon, 2006). Among them, the increase of IL-8 was the most significant. The lipopolysaccharide derived from Fn also promoted the secretion of MMP9 in macrophages, which may play a role in promoting tumor cell invasion and metastasis. Therefore, we speculate that the influence of Fn on the polarization of macrophages depends on the microenvironment, where the macrophages are located. In addition, not all components of Fn induce macrophage M2 polarization. Autoinducer-2 (AI-2) is a non-species-specific autoinducer that is involved in interspecies communication and is a main signal-type molecule of the quorum-sensing system in bacteria (Thompson et al., 2015). Wu and his colleagues found that AI-2 from Fn promoted macrophage M1 polarization *via* a mechanism involving TNFSF9/TRAF1/p -AKT/IL-1β signaling (Wu et al., 2019).

### 10.2 Fn and lymphocyte immunosuppression

A cross-sectional study of 598 CRC cases found that the amount of tissue Fn was inversely related to the density of CD3+ T cells in CRC tissue, but not to the density of FOXP3+ or CD8+ T cells (Mima et al., 2015). This is consistent with the research on murine model by Kostic et al (Kostic et al., 2013). Another cross-sectional study based on 933 CRC cases in two US-wide prospective cohort studies, showed an inverse association of Fn with the densities of CD3+ T cells and CD3+ CD4+ CD45RO+ memory helper T cells in CRC (Borowsky et al., 2021). Similarly, Fn was observed to associate with a lower density of CD8+ T cells in human CRC liver metastases (Sakamoto et al., 2021). Several studies have shown that Fn has immunosuppressive activity in lymphocytes. On the one hand, Fn can induce apoptosis of Jurkat T-cells (Jewett et al., 2000). On the other hand, Fn suppresses human T-cell activation by stopping cells in the mid-G1 phase of the cell cycle (Shenker and Datar, 1995). Additionally, Fn can inhibit human T-cell response to mitogens and antigens (Shenker and DiRienzo, 1984). Fap2 and RadD are large proteins localized to the outer membrane of Fn. The lymphocyte death induced by Fn mainly depends on Fap2 and RadD (Kaplan et al., 2010). Incubating lymphocytes with membranes carrying the Fap2 or RadD mutations resulted in partial decreases in cell death of 51% and 27%, respectively, whereas incubating lymphocytes with membranes containing the Fap2 and RadD double mutations led in a 77% reduction in cell death. Fn membranes containing Fap2 and RadD trigger cell death in lymphocytes at levels similar to incubation with whole cells, suggesting that the lethal action of Fn on lymphocytes is not energy-dependent. Natural killer cells (NK cells) are a type of cytotoxic lymphocyte that plays an important role in the body’s innate immune system. Tumors, viruses, parasites, and bacteria can be killed by NK cells directly or indirectly (Koch et al., 2013). T cell immunoglobulin and ITIM domain(TIGIT) is an inhibitory receptor present on human NK cells as well as a variety of T cells. The Fap2 protein on Fn interacts directly with TIGIT, inhibiting cytotoxicity of NK cells and T cells, weakening the killing effect on cancer cells (Gur et al., 2015). In addition, Fn also binds and activates the human inhibitory receptor CEA cell adhesion molecule 1(CEACAM1), thereby inhibiting the activity of T and NK cells (Gur et al., 2019). The trimeric autotransporter adhesin CbpF of Fn has been reported to bind specifically to CEACAM1 and mediate the inhibition of T Cell Function (Brewer et al., 2019; Galaski et al., 2021). Consistent with the result above, a study by Kim et al. showed that Fn inhibited the viability of NK cells *in vitro*, and reduced NK cell density in mouse intestinal tissues (Kim et al., 2021).

## 11 Fn affects CRC response to therapy

The 5-year survival rate in advanced CRC patients is less than 10% (Dahan et al., 2009). Chemotherapy is one of the effective treatments for patients with advanced CRC. Although most CRC patients initially respond to chemotherapy,cancer progression eventually occurs due to the drug resistance. Fn, as the intratumoral bacteria in CRC, is one of the driving factors of CRC resistance to chemotherapy. Autophagy can assist cells to cope with intracellular and external stressors such as hypoxia, nutritional deficiency, or cancer therapy, eventually promoting cancer development (Amaravadi et al., 2019). Fn promotes CRC chemoresistance by influencing autophagy (Yu et al., 2017). Fn targets TLR4/MYD88 signaling, leading to downregulation of miR-4802 and miR-18a. The loss of miR-18a and miR-4802 result in increased ULK1 and ATG7 gene expression, subsequently enhancing the activity of Autophagy, leading to CRC cells’ chemoresistance to Oxaliplatin and 5-FU. Baculoviral IAP repeat containing 3(BIRC3), as an inhibitor of apoptosis protein family member, mediates cell apoptosis resistance by inhibiting caspase cascade (Bertrand et al., 2008). Fn infection upregulates BIRC3 by activating TLR4/NF-κB pathway (Zhang et al., 2019). The NF-κB P65 enhances BIRC3 gene transcription by binding to its upstream promoter region. The upregulation of BIRC3 induced by Fn confers CRC cells chemoresistance to 5-Fu.

Immune checkpoint therapy activates antitumor immune response by blocking interactions between T cell inhibitory receptors and their cognate ligands, resulting in durable tumor regression. In a subset of patients, drugs of blocking immune checkpoints, such as programmed cell death protein 1 (PD-1) and its ligand PD-L1, have shown significant efficacy. However, anti-PD-1/PD-L1 therapy is deemed ineffective for the majority of CRC patients, and only those with high MSI-H and a high mutation burden respond to the treatment (Kreidieh et al., 2020). The presence of Fn in CRC tissue may be a double-edged sword. On the one hand, infection of high dose Fn can induce PD-L1 expression in CRC cells by activating the stimulator of interferon response cGAMP interactor 1 (STING) signaling. It is well known that PD-L1 mediates the immune escape of cancer cells. On the other hand, due to the upregulation of PD-L1, CRC is more sensitive to immune checkpoint blockade therapy. The study based on 41 CRC patients undergoing PD-1 blockade therapy, found that patients with Fn positive CRC had longer progression-free survival than Fn negative CRC. Experiments based on organoid and mouse subcutaneous homograft tumor model showed that Fn infection enhances the antitumor effect of PD-L1 blockade (Gao et al., 2021). This may provide us with a new perspective: whether Fn levels in CRC tissue can be used as an indicator for screening patients who potentially respond to immune checkpoint therapy. In addition, a study based on 1041 CRC patients shows that the presence of Fn was inversely associated with tumor-infiltrating lymphocytes(TIL) in MSI-H tumors, but positively correlated with TIL in non-MSI-H CRC (Hamada et al., 2018). We need further studies to determine whether the effect of Fn on lymphocytes depends on MSI status in CRC. If Fn can increase lymphocyte recruitment in patients with non-MSI-H CRC (account for nearly 85% of all CRC patients) for immune therapy.

## 12 Strategies for CRC prevention and treatment by anti-Fn

**Figure 4:**
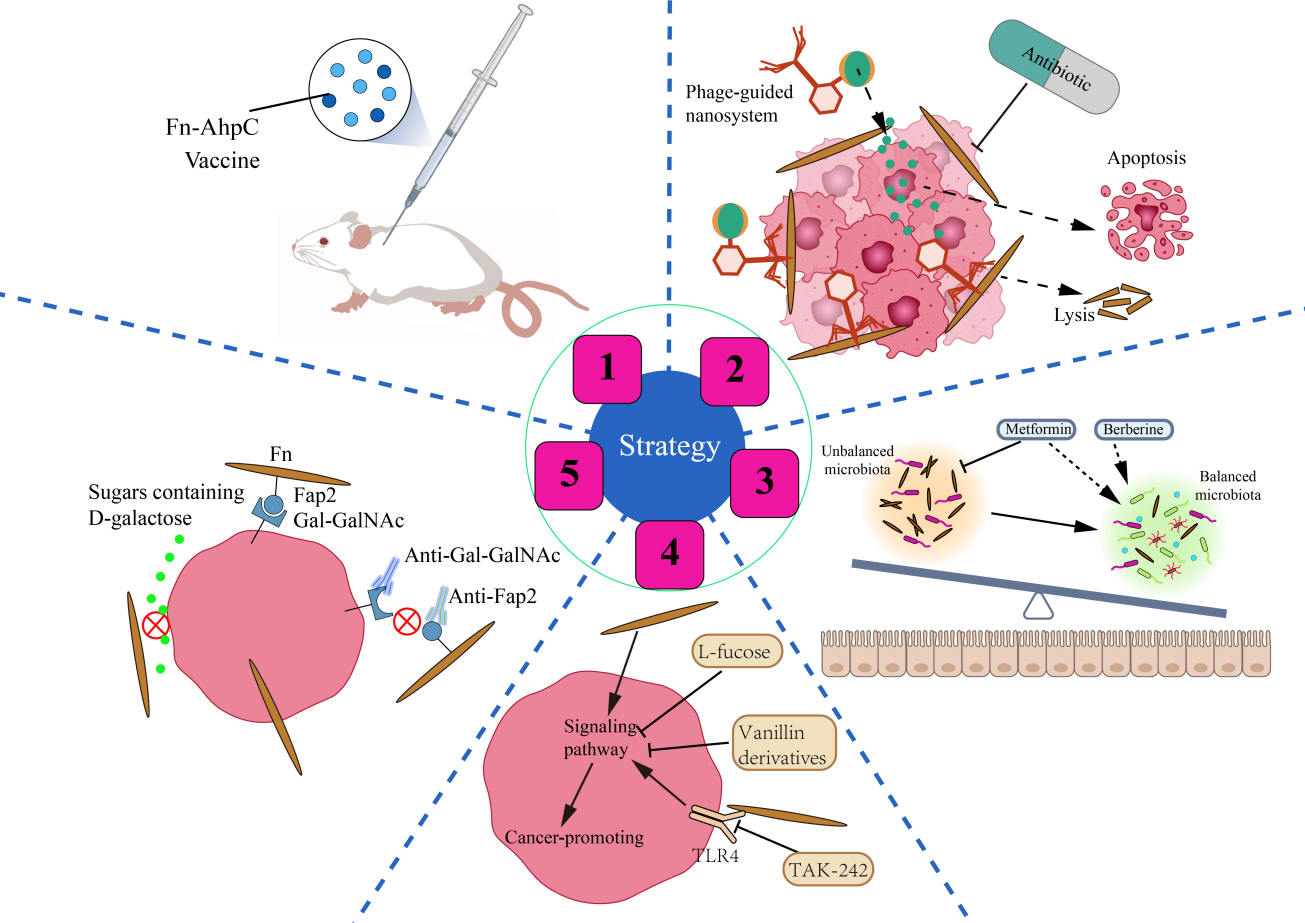
Strategies for CRC prevention and treatment by anti-Fn. 1: Prevention of Fn infection. Fn derived vaccine enhances the immunity of mice to Fn infection. 2: Inhibition/Elimination of intratumoral Fn. Antibiotics can significantly reduce the intratumoral Fn load. Phage guided nano system with drug can kill two birds with one stone in CRC. 3: Rebalance of gut microbiota. Some non-antibiotic drugs such as berberine and metformin can not only inhibit Fn but also improve gut flora. 4: Reverse Fn-activated signaling. A small part of compounds and molecular inhibitors such as L-fucose, vanillin derivatives, TAK-242 can selectively inhibit the Fn activated oncogenic signaling pathway. 5: Blockade of Fn’s cell adhesion and internalization. Antibodies such as antibodies targeting Gal-GalNAc or Fap2 and sugars such as D-galactose can block the adhesion of Fn to CRC cells, and eliminate the malignant effect of Fn from the source.

Given the deleterious effects of Fn in tumors of CRC patients, we still lack effective countermeasures. we should focus on how to accurately eliminate Fn and its adverse effects. At present, in this field, researchers have carried out various tentative studies (**Figure 4**).

### 12.1 Prevention of Fn infection and invasion

It is well known that we have successfully prevented Mycobacterium tuberculosis infection in humans by vaccination with Bacillus Calmette-Guérin (BCG). Similarly, can we prevent the invasion of Fn into the human body by vaccination? Guo et al. have found that the Fn-AhpC recombinant protein could be specifically recognized by antibodies present in the serum of CRC patients. Systemic prophylactic immunization with AhpC/alum significantly protected 77.3% of mice from infection (Guo et al., 2017). Such results are encouraging. Fn-derived proteins such as AhpC may serve as a potential vaccine candidate against Fn inhabitation or infection in the gut, which is of great significance for the prevention of CRC associated with Fn infection. Whether there is other antigen in Fn that is more suitable to be used as a vaccine still needs more exploration and verification by scientific researchers.

### 12.2 Inhibition/Elimination of intratumoral Fn

Bullman and colleagues treated mice bearing colon cancer xenografts with the antibiotic metronidazole and found that metronidazole treatment reduced intratumoral Fn burden and overall tumor growth significantly (Bullman et al., 2017). Although antibiotics such as metronidazole have a significant killing effect on Fn, they can also cause intestinal flora disturbance, affecting the colonization and activity of some probiotics. Phages can infect and lyse bacteria, replicate and degrade biofilm substrates, and are promising drugs for the treatment of bacterial infections (Agarwal et al., 2018). Since the attack mechanism of phages is highly specific to bacterial species, phage therapy should be particularly suitable for the accurate removal of tumor-promoting bacteria (Servick, 2016). Zheng et al. isolated a phage strain from human saliva that could specifically destroy Fn. They constructed a phage-guided biotic–abiotic hybrid nanosystem by using irinotecan(a first-line drug against CRC), dextran nanoparticles, and phages. This nanosystem exhibits powerful advantages, not only eliminating intratumoral Fn, reversing Fn-induced chemoresistance and enhancing chemoresponse, but also possessing remarkable tumor-specific targeting ability, thereby reducing the toxic and side effects of chemotherapeutic drugs. In addition, the system enhanced the proliferation of the anticancer Clostridium butyricum. Silver nanoparticles (AgNPs) exhibit excellent antibacterial activity but lack bacterial specificity. Dong et al. electrostatically assembled AgNPs on the surface of a specifically Fn-binding M13 phage (Dong et al., 2020). AgNPs were precisely directed to Fn in the tumor microenvironment due to the ability of phages to target Fn. AgNPs and M13 phage synergistically remove intratumoral Fn and reverse immunosuppressive TME, characterized by increased maturation of dendritic cells, M1 polarization of macrophages, and enhanced anti-tumor effect of T cells. Experiments in mouse allograft tumor models showed that it inhibited tumor growth and enhanced the therapeutic response to FOLFIRI chemotherapy and anti-PD-1 therapy.

### 12.3 Rebalance of gut microbiota

Fn gavage changes the structure of the intestinal microflora in mice and also promotes tumorigenesis (Yu et al., 2015). Fn may disturb the balance of gut microbiota by increasing opportunistic pathogens and reducing probiotics. Therefore, the disordered gut microbiota and the enrichment of Fn in the intestinal lumen and tumor tissue of patients with CRC may be mutually causal. Berberine, an isoquinoline alkaloid, is the pharmacological component of the Chinese herbal medicine Coptis Rhizoma, which has been used in China for thousands of years to treat intestinal infections, especially bacterial diarrhea (Tang et al., 2009). Berberine not only reverses the microbiota imbalance induced by Fn gavage but also downregulates mucosal immune cytokine secretion and activation of JAK/STAT and MAPK/ERK pathways (Yu et al., 2015). This implies the potential value of berberine in CRC treatment, especially in people with high levels of Fn. A study published in 2018 screened more than 1,000 non-antibiotic drugs for their effects on gut bacteria and found that metformin significantly inhibited Fn at colonic concentrations of about 1.5 mM (Maier et al., 2018). Given this, Huang et al. conducted a study and found that metformin reduced the Fn abundance, alleviated symptoms caused by Fn, and rescued Fn-induced tumorigenicity in APC^Min/+^ mice (Huang et al., 2020). Drugs such as metformin, which both inhibit specific pathogens and regulate intestinal flora disturbances, may maximize the benefits of treatment, especially in those with CRC who have diabetes. Another advantage that cannot be ignored is that these drugs have been used in clinics, and their side effects and adverse reactions have been mastered by people.

### 12.4 Reverse Fn-activated signaling

The fourth strategy is to target Fn-activated signaling pathways and reverse Fn-induced malignant transformation. L-fucose is a naturally occurring monosaccharide found in food or the body. Duan et al. revealed that L-fucose reversed the tumor-promoting properties of Fn by inhibiting its ability to activate jak-stat3 signaling and EMT in colon cancer cells *in vitro (* Duan et al., 2021). Despite the lack of support from *in vivo* research data and rigorous validation of the mechanism, this finding is still eye-catching. Zhou et al. found that vanillin derivatives IPM711 and IPM712 reversed Fn-induced proliferation and migration of CRC by inhibiting the activation of the E-Cadherin/β-Catenin pathway (Zhou et al., 2022). IPM712 has been reported to show better anticancer activity than 5-Fu with low toxicity at therapeutic concentrations (Ma et al., 2020). TLR4 is a key receptor that mediates the tumor-promoting effect of Fn. Therefore, TLR4 inhibitor TAK-242 is a potential effective treatment drug for Fn-infected CRC patients (Chen et al., 2017; Chen et al., 2018).

### 12.5 Blockade of Fn’s adhesion and internalization for CRC cell

The promoting effect of Fn on the malignant behavior of CRC cells is mainly based on the high adhesion and invasiveness of Fn to CRC. As described in the previous, the molecular basis of Fn adhesion and invasion is the specific binding of lectin Fap2 and adhesins FadAc to Gal-GalNAc and E-cadherin respectively. We speculate that it is promising to develop antibodies targeting Gal-GalNAc or Fap2 to selectively block Gal-GalNAc/Fap2 binding, because Gal-GalNAc is highly expressed in CRC cells and low or not expressed in non-CRC cells. Furthermore, it has been reported that sugars containing D-galactose can inhibit Fn adhesion and invasion of CRC cells significantly (Abed et al., 2016). However, whether oral administration of sugars containing D-galactose reverses the cancer-promoting effect of Fn remains to be further investigated.

## 13 Conclusion and future direction

In the past ten years, from the appearance to the cause and mechanism, the understanding of Fn as a “driver” of CRC development has gradually become clear. However, the role and mechanism of Fn in the initial stage of CRC remain unknown for us. CRC includes various pathological subtypes and molecular subtypes. To our knowledge, different CRC cell lines have different responses to Fn infection. The question that the malignant biological behavior of which subtype of CRC is aggravated most obviously by Fn infection deserves further study. It will help us accurately identify which subgroup of patients in risk needs to be intervened if Fn is present in their tumors. In addition, we need to determine the threshold value of bacterial load in tumor, beyond which, biological intervention is required for patients. As the previous studies have confirmed that the promoting effect of low-dose Fn infection on the malignant biological behavior of CRC cells is weak. If we use drugs to interfere with them excessively, the harmful side effects may be far greater than the therapeutic effects.

Previous studies on the effect of Fn in the tumor immune microenvironment mainly involve monocytes, macrophages, T cells and NK cells. How other components of the tumor microenvironment, such as tumor-associated fibroblasts, MDSCs, and dendritic cells, respond to Fn infection remain covered. The effect of Fn on macrophage polarization is still controversial. Whether macrophages are polarized to cancer-promoting or cancer-suppressing direction may depend not only on Fn, but also on the components of microenvironment. The current mechanism studies on Fn and TAM are based on animal cells or animal models or human leukemia cell models, which are far from the real state of TAM in human CRC. In addition, most of the current studies on Fn and the tumor immune microenvironment are binary studies, and we should consider their responses to Fn invasion as a whole. CRC contains multiple subtypes. They have different immune cell infiltration profiles. Previous studies on Fn and tumor immunity ignored the molecular subtypes of CRC. We need to determine whether the effects of Fn on immune cells are consistent among various CRC subtypes. This will provide reference for the determination of clinical immunotherapy strategy.

We have already uncovered part of mechanisms of Fn in CRC exacerbation. Next, we should pay more attention to translating these mechanisms into clinical applications. In terms of disease control strategies, prevention is better than treatment. As a prevention method, immunization with antigen derived-from Fn deserves further exploration. Since Fn activates multiple cancer promoting signal pathways, we advise researchers to try to use small molecule inhibitors to reverse the harmful effects of Fn infection on CRC. Besides, Nano-systems coupled with chemotherapeutics are very promising, as not only enhance the tumor-targeting ability of chemotherapy but also reverse Fn-induced resistance and immunosuppression. In view of the immunosuppressive effect of Fn on lymphocytes, we believe that TIGIT and PD-L1 dual immune checkpoint blockade therapy may have better efficacy in patients with high intratumoral Fn levels. In addition, we should also consider introducing probiotics to antagonize Fn and activate anti-tumor immune responses, such as Lactobacillus rhamnosus and Bifidobacterium Breve (Yoon et al., 2021; Si et al., 2022).

## Author contributions

All authors contributed to the article and approved the submitted version. SO wrote the manuscript draft. HW, YT were responsible for the literature search. SO and SR drew the figures, JY and KL were responsible for language polishing. ZG, YW and HH contributed to furtherly editing the manuscript. RH provided direction and instruction and revised the manuscript.

## Funding

This work was supported by National Natural Science Foundation of China under Gant [No. 81872034], the Natural Science Foundation of Heilongjiang Province under Gant [No. H2017016], Wu Jieping Medical Foundation under Gant [No. 320.6750.19092-41], Chen Xiao-ping Foundation for The Development of Science and Technology of Hubei Province under Gant [No. CXPJJH12000002-2020025].

## Acknowledgments

Thanks to Yifan Mu, School of Architecture, Harbin Institute of Technology, Harbin, China, for her assistance in pictures.

## Conflict of interest

The authors declare that the research was conducted in the absence of any commercial or financial relationships that could be construed as a potential conflict of interest.

## Publisher’s note

All claims expressed in this article are solely those of the authors and do not necessarily represent those of their affiliated organizations, or those of the publisher, the editors and the reviewers. Any product that may be evaluated in this article, or claim that may be made by its manufacturer, is not guaranteed or endorsed by the publisher.

## Glossary

**Table:**

CRC	Colorectal cancer
Fn	Fusobacterium nucleatum
APC	Adenomatous polyposis coli
MSI	Microsatellite instability
CMS	Consensus molecular subtypes
SPF	Specific-pathogen-free
GF	Germ-free
Gal-GalNAc	D-galactose-b(1-3)-N-acetyl-D-galactosamine
IL6	Interleukin 6
IL8	Interleukin 8;
COX2	Cytochrome c oxidase subunit II
TNF-a	Tumor necrosis factor a
MIP3a	Macrophage Inflammatory Protein-3
CCL20	C-C motif chemokine ligand 20
IL22	Interleukin 22
IL21	Interleukin 21
IL17F	Interleukin 17F
AOM	Azoxymethane
DSS	Dextran sodium sulfate
RASA1	RAS p21 protein activator 1
MAPK	Mitogen-activated protein kinase
PAK1	P21-activated kinase 1
CXCL1	C-X-C motif chemokine ligand 1
CXCL16	C-X-C motif chemokine ligand 16
CXCR6	C-X-Cmotif chemokine receptor 6
KRT7	Keratin 7
CARD3	Caspase activation and recruitment domain 3
EMT	Epithelialmesenchymaltransition
METTL3	N6-adenosinemethyltransferase complex catalytic subunit
ICAM1	Intercellular adhesion molecule 1
AhR	Aryl hydrocarbon receptor
CSC	Cancer stem cell
MDM2	Mouse double minute 2 homolog
ALDH	Aldehyde dehydrogenase
OCT4	Octamer-binding transcription factor 4
ATP	Adenosine triphosphate
ENO1	Enolase 1
H3K27Ac	Acetylation of histone H3 lysine 27
ANGPTL4	Angiopoietin-like 4
CYP2J2	Cytochrome P450 2J2
EpOME	Epoxyoctadecaenoic acids
TME	Tumor microenvironment
TAMs	Tumor-associated macrophages
MDSCs	Myeloid-derived suppressor cells;
CCL3	C-C motif chemokine ligand 3
CXCL2	C-X-C motif chemokine ligand 2
CCL5	C-C motif chemokine ligand 5
MMP9	Matrix metallopeptidase 9
FOXP3	Forkhead box P3
CEACAM1	CEA cell adhesion molecule 1
TIGIT	T cell immunoglobulin and ITIM domain
PD-1	Programmed cell death protein 1
PD-L1	PD-1 ligand 1
